# Utilization of varenicline among Kansas Medicaid enrollees

**DOI:** 10.1371/journal.pone.0333723

**Published:** 2025-10-07

**Authors:** Nathalia M. Machado, Aaron J. Katz, Kimber P. Richter, Edward F. Ellerbeck

**Affiliations:** 1 TSET Health Promotion Research Center, Stephenson Cancer Center, The University of Oklahoma Health Campus, Oklahoma City, Oklahoma, United States of America; 2 Department of Population Heath, University of Kansas Medical Center, Kansas City, Kansas, United States of America; Auburn University, UNITED STATES OF AMERICA

## Abstract

**Background:**

Smoking prevalence among Medicaid beneficiaries is twice that of privately insured adults, yet effective smoking cessation treatments remain underutilized. Understanding varenicline use, the most effective smoking cessation medication, can inform efforts to improve tobacco treatment for Medicaid enrollees.

**Objectives:**

To describe varenicline utilization among Kansas Medicaid (KanCare) enrollees from 2014–2021, including changes related to the FDA’s December 2016 removal of the black box warning, and explore changes and demographic differences in its use.

**Research design:**

This retrospective cohort study analyzed pharmacy claims data for varenicline utilization from January 1, 2014, to December 31, 2021. Tobacco use was estimated from the KanCare External Quality Review survey.

**Subjects:**

The sample included 78,295 new adult enrollees (age 18+) with at least 11 months of continuous coverage.

**Measures:**

The primary outcome was varenicline utilization, defined as any pharmacy claim for the medication. Secondary outcomes included time to first prescription, completion of the 12-week treatment, and demographic differences in use.

**Results:**

With an estimated 30% smoking prevalence, only 12.7% of KanCare smokers received varenicline, and just 1.6% completed a full course of treatment. Among 78,295 new enrollees, 2,980 (3.8%) had one or more claims for varenicline, with the mean time from enrollment to first prescription being 1.5 years (SD 1.3). Utilization was higher among males (4.8%) than females (3.4%) and varied by race/ethnicity (Whites: 4.4%, Blacks: 2.7%, American Indians: 2.5%, Hispanics: 1.6%, p<0.0001). Among those who initiated treatment, only 13.5% (N=380) completed the recommended 12-week regimen.,

**Conclusions:**

Despite the high smoking prevalence among KanCare beneficiaries, varenicline is underutilized, with significant demographic disparities. Men and non-Hispanic Whites were more likely to receive varenicline than women and other racial and ethnic groups, despite comparable or higher smoking rates among other subgroups. Medicaid programs must intensify efforts to provide equitable and effective tobacco treatment.

## Introduction

The prevalence of cigarette smoking among Medicaid enrollees is double that of privately insured adults [1]. Medicaid spends $40 billion to treat smoking-related diseases, including several types of cancers, cardiovascular diseases, chronic obstructive pulmonary disease (COPD), diabetes, and other chronic conditions, accounting for over 15% of Medicaid’s total annual spending [2]. Treating tobacco dependence is the most effective way to reduce this burden, and there are proven treatments to help people quit, including pharmacotherapy and behavioral support.

Seven medications are currently approved by the U.S. Food and Drug Administration (FDA) to assist people in quitting smoking, including bupropion, varenicline, and five forms of nicotine replacement therapy (NRT). Most (43) state Medicaid programs cover all seven FDA-approved cessation medications as of December 2022 [3]. Combining pharmacotherapies increases the odds of quitting, and the combination of counseling and medication provides the best cessation outcomes [4,5]. However, when a combination is not possible, successful quitting with a single medicine is best with varenicline. Varenicline reduces cravings for smoking by blocking the pleasant effects of nicotine on the brain and is the most effective monotherapy for smoking cessation [4].

Despite its effectiveness, an analysis of 2010–2013 claims estimated that only one in ten Medicaid enrollees who smoked received cessation pharmacotherapy, with five states, including Kansas, showing utilization of 3% or lower [6]. Varenicline is the single most effective smoking cessation medication [7], but early reports of cardiovascular and neuropsychiatric adverse events led to an FDA black box warning. [8] Subsequent studies, however, demonstrated its safety in patients with stable psychiatric disease, leading to the removal of the black box warning in 2016 [9].

Understanding how varenicline has been used over time in Medicaid populations can inform efforts to reduce tobacco-related morbidity, mortality, and healthcare costs. Low utilization may reflect missed opportunities to support cessation among populations with high smoking prevalence and disproportionate disease burden. To explore this further, we examined detailed enrollee-level Medicaid claims data for varenicline utilization in Kansas from 2014 to 2021, before and after the FDA’s removal of the black box warning.

## Methods

### Data

We used Medicaid enrollee and pharmacy extract files to assess claims from 2014 to 2021, focusing on claims for varenicline. We focused on varenicline because it is only available by prescription, making it more reliably captured in pharmacy claims data compared to over-the-counter nicotine replacement therapies (NRT) or counseling, which are often not billed for or captured in administrative claims.

**Fig 1:**
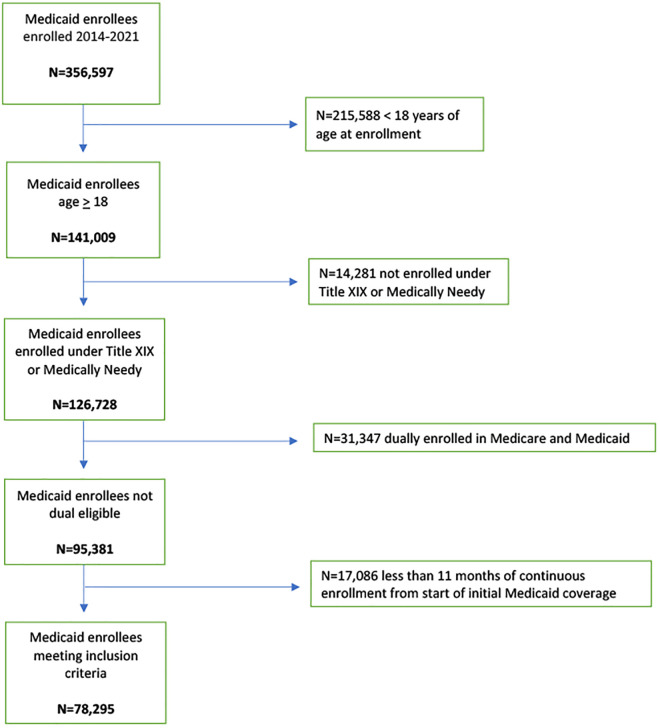
Selection of eligible Medicaid beneficiaries.

To create our analytic dataset, we merged two distinct Medicaid files: the beneficiary enrollment database and the pharmacy claims database. The enrollment database includes demographic and enrollment history information for all Medicaid beneficiaries. The pharmacy claims database contains detailed records of outpatient prescription medications that were dispensed and reimbursed by Medicaid. The resulting analytic dataset combines person-level demographic, enrollment, and prescription drug information, allowing for longitudinal tracking of varenicline use among enrollees. This dataset was de-identified and accessed during the analysis period (May 2023–October 2024). We limited our cohort to adults 18 or older who were enrolled under “Medically Needy” or “Title XIX” categories in their first enrollment period. We excluded certain Medicaid enrollment categories because they are tied to specific time periods and can be time-limited (e.g., pregnant women), making it unfeasible to track claims over-time. We also excluded beneficiaries who enrolled before 2014, as our dataset began in 2014 and claims prior to their enrollment could not be captured. Additional exclusions included individuals with dual eligibility in Medicaid and Medicare (because for dual enrollees, Medicare serves as the primary payer, and related claims would not be available in this dataset), as well as those with less than 11 months of continuous enrollment from the initial Medicaid coverage period, to avoid including individuals with only brief coverage periods. These criteria resulted in a sample of 78,295 beneficiaries (Fig 1).

To determine annual use rates, we conducted a secondary analysis limited to beneficiaries who were continuously enrolled in Medicaid for at least 11 months in each calendar year of the study period (mean sample of 21,286 beneficiaries enrolled per year, SD 9,181). This criterion was applied to ensure consistent coverage and reduce bias from partial-year enrollment, which could result in incomplete data on medication use.

The study was reviewed and approved under the University of Kansas Medical Center Human Research Protection Program (HRPP) (IRB S TUDY0015000).

### Measure

The primary outcome was varenicline use, operationalized as having any claim for varenicline filled between 2014–2021. Demographic characteristics (age, sex, race and ethnicity) and enrollment information (dates of enrollment, eligibility category, and dual eligibility) were assessed through the enrollee file. National Drug Codes, prescription fill date, quantity dispensed, and days of supply were assessed through the pharmacy extract file.

### Analysis

We identified the proportion of enrollees who had claims for varenicline at any time during 2014–2021, overall, by year, and by selected demographic characteristics. We compared varenicline use rates by gender, race, and ethnicity using the chi-square test, which generated p-values to assess the association between these variables. In addition, we conducted a multivariable logistic regression analysis to examine the adjusted associations between demographic characteristics and the likelihood of varenicline use, controlling for age at enrollment, sex, race/ethnicity, and Medicaid eligibility category.

We also calculated the number and proportion of enrollees who received a total quantity of varenicline through claims sufficient to cover at least 84 days of treatment, consistent with the recommended 12-week course outlined in clinical guidelines [10,11]. A gap of more than 14 days between the end of supply from the last fill and a subsequent prescription was considered to be a new course of treatment. We selected this 14-day threshold based on the recommendation for dosing titration in most individuals initiating varenicline; after beginning varenicline use, those who miss taking the medication for several days or longer likely need to re-start the process and begin again at the lower initial dose. Furthermore, shorter gaps (e.g., ≤ 14 days) are more likely to signal delays in refilling the medication, whereas longer gaps are more likely to reflect meaningful interruptions or discontinuation of therapy (e.g., due to adverse effects).

To assess potential changes in varenicline use following the FDA’s removal of the black box warning in December 2016, we examined dispensing rates over time and conducted two complementary statistical analyses. First, we performed an interrupted time series (ITS) analysis using segmented regression of monthly dispensing rates per 1,000 non-dual eligible beneficiaries. This approach evaluated changes in both the level and slope of the dispensing trend before and after December 2016. Second, we conducted a two-sample t-test comparing the mean monthly dispensing rates during the 12 months before versus the 12 months following the policy change, providing an overall assessment of differences between the two periods.

To understand the link between enrollment time and varenicline utilization, we calculated the number of days from first enrollment in Medicaid until receipt of their first claim for varenicline, censored at three years post-enrollment. Analyses were performed using R (version 4.0.2).

Since Medicaid does not routinely collect enrollees’ smoking status, we estimated that 30% of adult Medicaid recipients in Kansas were smokers based on the 2021 KanCare External Quality Review survey [12]. We acknowledge this as a limitation, as the Medicaid data do not provide the denominator needed to estimate variability around the smoking prevalence. EQRO survey smoking estimates from 2014 to 2021 ranged from 30% to 33%. To address uncertainty in these estimates, we conducted a sensitivity analysis using this range of smoking prevalence values to express varenicline utilization under different assumptions. Table 2 presents these estimates, allowing for a more transparent assessment of potential variation in utilization rates.

**Table 2:** Varenicline use estimates across a range of smoking prevalence assumptions.

	Smoking prevalence from EQRO surveys (%)	Estimated # of smokers (N = 78,295)	% with any varenicline dispensed (N = 2,980)	% completing 12-week of treatment (N = 380)
2014	33.45	26,190	11.38	1.45
2015	32.21	25,219	11.82	1.51
2016	33.18	25,978	11.47	1.46
2017	31.87	24,953	11.94	1.52
2018	31.76	24,866	11.98	1.53
2019	30.03	23,512	12.67	1.62
2020	30.28	23,708	12.57	1.60
2021	30.30	23,723	12.56	1.60

## Results

### Sample characteristics

From January 2014 to December 2021, we identified 78,295 unique adult Medicaid enrollees (Fig 1). Their mean age at first enrollment was 35.6 years (SD 12.5), and the average duration of KanCare coverage was 37.1 months (SD 24.9). Demographic characteristics are presented in Table 1.

**Table 1:** Demographic characteristics of Kansans Medicaid enrollees and individuals dispensed varenicline for smoking cessation between 2014 and 2021.

Characteristics*		No. (%)	No. (%)	–	–
		Total N = 78,295	With at least 1 dispensing of varenicline N = 2,980	% with ≥1 claims	*p-value*
**No.**	Total sample	78,295	2,980	3.81	–
	Estimated smokers (30%)	23,489	2,980	12.69	–
**Age; Mean (SD)**					
	At first enrollment date;	35.63 (12.5)	40.71 (10.9)	–	–
	At first Chantix dispensing date	–	41.73 (10.9)	–	–
**Sex**	Female	57,448 (73.4)	1,976 (66.3)	3.44	<0.0001
	Male	20,847 (26.6)	1,004 (33.7)	4.82	
**Race**	American Indian	1,181 (1.5)	30 (1.01)	2.54	<0.0001
	Black or African American	10,011 (12.8)	272 (9.1)	2.72	
	Other/Unknow	10,445 (13.3)	172 (5.7)	1.65	
	White	56,658 (72.4)	2,506 (84.1)	4.42	
**Ethnicity**	Hispanic, Spanish, or Latinx	5,778 (7.4)	95 (3.2)	1.64	<0.0001
	Not Hispanic, Spanish, or Latinx	72,517 (92.6)	2,885 (96.8)	3.98	
**Medicaid category**	Medically Needy	4,874 (6.2)	159 (5.3)	3.26	0.04
	Title XIX	73,421 (93.8)	2,821 (94.7)	3.84	

### Varenicline utilization

Among all enrollees, 2,980 (3.8%) had one or more claims for varenicline. Assuming that 30% of enrollees smoke, 12.7% of all Medicaid smokers had at least one varenicline prescription filled.

Table 2 shows varenicline use estimates across years under varying smoking prevalence assumptions from the annual EQRO survey. Varenicline utilization rates ranged from 11.38% to 12.67% across these estimates, and completion of the 12-week treatment course ranged from 1.54% to 1.62%. The highest estimated utilization occurred under the study’s 30% smoking prevalence assumption (12.67%), while the lowest occurred in 2014 when the smoking prevalence was highest (33.45%), yielding an estimated use rate of 11.38%. These variations highlight the modest influence of smoking prevalence assumptions on calculated utilization rates.

**Fig 2:**
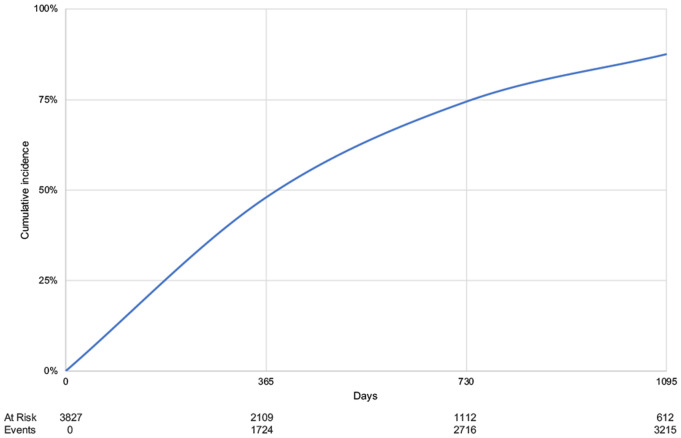
Cumulative incidence of time of enrollment in Medicaid before first varenicline dispensing.

On average, beneficiaries were enrolled in Medicaid for 1.5 years (SD 1.3) before receiving varenicline. Fig 2 shows the cumulative incidence of varenicline utilization during the first three years of Medicaid enrollment.

Males were more likely than females to receive varenicline (4.8% vs. 3.4%, respectively; P < 0.001). Whites (4.4%) were more likely to receive varenicline than Blacks (2.7%), American Indians (2.5%), Hispanic (1.6%), and Others (1.6%) (P < 0.001) (Table 1). These disparities remained significant in adjusted analyses: males had higher odds of varenicline use than females (aOR: 1.11, 95% CI: 1.02–1.20, p = 0.012), and individuals identifying as Black (aOR: 0.56, 95% CI: 0.49–0.64, p < 0.001), American Indian (aOR: 0.55, 95% CI: 0.37–0.78, p = 0.001), Hispanic (aOR: 0.46, 95% CI: 0.37–0.56), or Other/Unknown race (aOR: 0.35, 95% CI: 0.30–0.41, p < 0.001) had significantly lower odds of varenicline use compared to White or non-Hispanic individuals (Table 3).

**Table 3:** Adjusted associations between demographics and varenicline use.

Variable		aOR	95% CI	*p-value*
Age		1.04	1.04–1.04	<0.001
Sex	Female		Reference	
	Male	1.11	1.02–1.20	0.01
Race	White		Reference	
	Black or African American	0.56	0.49–0.64	<0.001
	American Indian	0.55	0.37–0.78	0.001
	Other/Unknown	0.35	0.30–0.41	<0.001
Ethnicity	Not Hispanic, Spanish, or Latinx		Reference	
	Hispanic, Spanish, or Latinx	0.46	0.37–0.56	<0.001
Category	Medically Needy		Reference	
	Title XIX	0.40	0.33–0.47	<0.001

### Patterns of varenicline use

Among those who initiated treatment, 13.5% (N = 380) received a sufficient quantity of varenicline claims to complete a standard 12-week course of treatment. This correlates with an estimated 1.6% of people who smoke in KanCare completing 12-weeks of varenicline treatment. Of the 2,980 that received varenicline, 1,083 (36.3%) initiated varenicline one or more additional times during their enrollment in Medicaid.

### Annual rates of use

**Fig 3:**
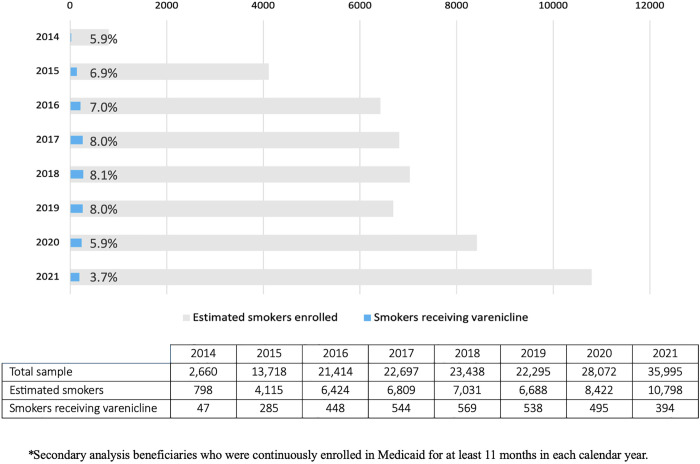
Varenicline dispensing among estimated smokers per year.

**Fig 4:**
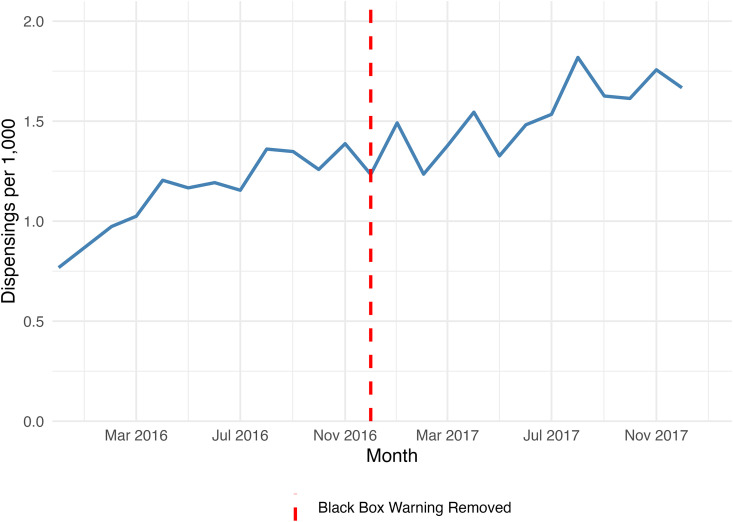
Monthly varenicline dispensing rates before and after the FDA black box warning removal in December 2016 (per 1,000 non-dual beneficiaries).

Among all adult enrollees, an average of 2.0% received varenicline in any given year from 2014–2021. Assuming a 30% smoking prevalence, an average of 6.5% of people who smoke received varenicline per year, ranging from 8.1% in 2018 to 3.7% in 2021 (Fig 3). Regarding the FDA’s removal of the black box warning for varenicline at the end of 2016, we conducted an additional analysis to evaluate potential changes in utilization. Results from the ITS indicated a significant upward trend in dispensing before December 2016 (p < 0.001). After the removal of the black box warning, the trend flattened, with no significant change in slope (p = 0.27) (Fig 4).

The t-test comparing monthly dispensing rates in the 12 months before versus after December 2016 showed a significantly higher mean in the post-removal period (1.52 vs. 1.14 per 1,000; p < 0.001), reflecting increases later in the post-removal period.

Notably, annual utilization rates declined from 8.0% in 2019 to 5.9% in 2020 and further to 3.7% in 2021, which may reflect COVID-19–related disruptions in healthcare access, prescribing practices, or patient engagement.

## Discussion

Despite smoking’s significant health impact, only a small proportion of KanCare enrollees receive varenicline to quit smoking, with consistently low utilization rates over the eight years analyzed. There was little change in varenicline utilization after the FDA ‘black box’ warning removal in 2016 [9]. Formal statistical testing of the FDA’s removal of the black box warning in 2016 showed a gradual increase in rates prior to the removal of black box warning, and modestly statistically significantly higher rates following the policy change. These findings suggest that the warning removal may have influenced utilization patterns in the short term, but changes in subsequent years suggest the importance of other potential factors. These data further suggest gender, racial, and ethnic disparities in varenicline utilization, delays in treatment initiation, and widespread failure to complete a full course of therapy.

Nationally, Medicaid coverage and utilization of tobacco cessation medications vary considerably across states and insurance types. According to recent CDC data [3], while some states have expanded comprehensive, barrier-free Medicaid coverage for cessation treatment, many continue to impose restrictions such as prior authorization requirements, limits on treatment duration, and caps on the number of covered quit attempts. A state-level analysis [13] of Medicaid claims data found that, on average, only 9.4% of Medicaid enrollees who reported a past-year quit attempt had claims for cessation medications. This rate ranged from just 0.2% in Arkansas to 32.9% in Minnesota, with 13 states reaching rates of 10% or higher—comparable to the rate observed in Kansas in this study.

Among these adult Medicaid enrollees, men and non-Hispanic Whites were more likely to receive varenicline than women and other racial and ethnic groups. These differences may be attributable to differences in smoking rates among subgroups of Medicaid enrollees, but overall, in Kansas, smoking rates among men, women, non-Hispanic Whites and Latinos are roughly comparable, and smoking among American Indians and Blacks exceed smoking rates in other racial groups [14]. While clinical appropriateness may vary by demographic group, varenicline has been shown to be effective across populations, supported by clinical trials and meta-analyses [15] demonstrating that it improves cessation rates across sexes, age groups, races/ethnicities, and socioeconomic levels, with no major subgroup differences in efficacy. Therefore, differences in utilization likely reflect factors other than efficacy, such as access, patient preferences, or prescribing practices. Furthermore, our findings are consistent with others showing substantially higher delivery of smoking cessation treatment among non-Hispanic Whites [16], including studies among Medicaid recipients [17].

Medicaid enrollees waited an average of 18 months before receiving varenicline. While clinical decisions should consider medical appropriateness, this delay in initiation of cessation therapy places these individuals at risk of ongoing smoking-related morbidities. Furthermore, many adults may lose Medicaid coverage before receiving the benefits of this smoking cessation treatment. Although COVID-19-related changes to Medicaid enrollment temporarily decreased the number of enrollees who lost coverage, those changes have since expired, and half of adult Medicaid recipients lose coverage within a year of enrollment [18].

Longer duration of varenicline use is associated with increases in long-term abstinence rates [19], but only a small proportion of varenicline recipients in this study received varenicline for the recommended 12-week treatment. To optimize varenicline effectiveness, additional strategies are needed to enhance adherence. Recent research highlights several promising approaches to support medication adherence, such as behavioral support and contingency management [20]. Monitoring adherence is a critical mediator of treatment outcome and should also be a standard component of smoking cessation treatment research.

### Limitations

The findings in this report are subject to at least four limitations. First, Medicaid claims data do not include smoking status, and we had to estimate the smoking percentage based on the statewide smoking prevalence among Medicaid enrollees. However, we incorporated a range of annual smoking prevalence estimates among Medicaid enrollees using state surveillance data, which showed slight variability in varenicline utilization estimates depending on the prevalence assumed. Regardless, the absence of individual-level smoking status remains an inherent limitation of using claims data. Second, claims data show how many prescriptions were filled, and the quantity dispensed, but do not reveal whether the patient took the medication, so our data may overestimate actual utilization. Third, we limited our analysis to beneficiaries enrolled at least 11 months, so we cannot assess varenicline use among those who entered and exited Medicaid enrollment during a single calendar year. Lastly, although varenicline is the single most potent cessation treatment, this study only examined varenicline use and did not include other forms of smoking cessation support, such as nicotine replacement therapy. Future analyses will aim to include the full range of cessation treatments to provide a more comprehensive understanding of treatment utilization patterns.

### Implications

Tobacco remains a major cause of illness and death among Medicaid enrollees. Varenicline is currently the most effective single agent for smoking cessation and these data suggest an urgent need for additional efforts to increase varenicline utilization, support adherence, and provide timely care. Medicaid services in Kansas, and most other states, are provided through managed care organizations that are required to monitor and report on the quality of their services. Most programs fail to collect smoking status which is a major barrier to quality assessment or improvement. To better serve patients, Medicaid programs should leverage their claims data to monitor and improve efforts to deliver high-quality care to those who smoke.
